# Molecular characterization of a complex small supernumerary marker chromosome derived from chromosome 18p: an addition to the literature

**DOI:** 10.1186/s13039-020-00519-w

**Published:** 2021-01-20

**Authors:** Eleonora Marchina, Michela Forti, Mariella Tonelli, Stefania Maccarini, Francesca Malvestiti, Chiara Piantoni, Elena Filippini, Elisa Fazzi, Giuseppe Borsani

**Affiliations:** 1 Laboratory of Cytogenetics and Molecular Genetics, Department of Molecular and Translational Medicine, University of Brescia, Brescia, Italy; 2 Unit of Child Neurology and Psychiatry, Civil Hospital, Department of Clinical and Experimental Sciences, University of Brescia, Brescia, Italy; 3 Division of Biology and Genetics, Department of Molecular and Translational Medicine, University of Brescia, Brescia, Italy; 4 TOMA Advanced Biomedical Assay, Busto Arsizio, Varese Italy

**Keywords:** Trisomy 18p, sSMC, a-CGH, Intellectual disability, Dysmorphisms

## Abstract

**Background:**

Small supernumerary marker chromosomes (sSMC) are a heterogeneous group of structurally abnormal chromosomes, with an incidence of 0,044% in newborns that increases up to almost 7 times in developmentally retarded patients. sSMC from all 24 chromosome have been described, most of them originate from the group of the acrocentric, with around half deriving from the chromosome 15. Non-acrocentric sSMC are less common and, in the 30 percent of the cases, are associated with phenotypic effect. Complex sSMC consist of chromosomal material derived from more than one chromosome. Genotype–phenotype correlations in patients with sSMC are difficult to assess. Clinical features depend on factors such as its size, genetic content, the involvement of imprinted genes which may be influenced by uniparental disomy and the level of mosaicism. Trisomy of the short arm of chromosome 18 (18p) is an infrequent finding and does not appear to be associated with a specific syndrome. However, mild intellectual disability with or without other anomalies is reported in almost one-third of the patients.

**Case presentation:**

Here we present clinical and molecular characterization of a new case of de novo complex sSMC consisting of the entire short arm of chromosome 18p associated with a centromere of either chromosome 13 or 21, evidenced in a 5-year-old boy during diagnostic workup for moderate intellectual disability and dysmorphisms. To date, only seven cases of isolated trisomy 18p due to a sSMC have been reported, three of which have been characterized by array CGH. In two of them the breakpoints and the size of the duplication have been described. In the manuscript we also reviewed cases reported in the DECIPHER database carrying similar duplication and also considered smaller duplications within the region of interest, in order to evaluate the presence of critical regions implicated in the pathological phenotype.

**Conclusions:**

Our case provides additional information about phenotypic effects of pure trisomy 18p, confirms chromosomal microarray analysis as gold standard to characterize complex sSMC, and supplies additional elements for genetic counselling.

## Background

Small supernumerary marker chromosomes (sSMC) are a heterogeneous group of structurally abnormal chromosomes, with an incidence of 0.044% in newborns that increases up to almost 7 times in developmentally retarded patients [1]. sSMC derived from all 24 chromosome have been described, the majority of them derive from the group of the acrocentric, with around half originating from chromosome 15 [2, 3]. About 30 percent of all sSMC are parentally inherited [4]. Non-acrocentric sSMC are less common and, in 30 percent of the cases, are associated with phenotypic effects [5]. Complex sSMC consist of chromosomal material derived from more than one chromosome.

Genotype–phenotype correlation for sSMCs is a challenging because clinical features depend on their size, the genetic content, the involvement of imprinted genes which may be influenced by uniparental disomy (UPD) and the level of mosaicism [6].

Trisomy of the short arm of chromosome 18 (18p) is an infrequent finding and does not appear to be associated with a specific syndrome. However, mild intellectual disability with or without other anomalies is reported in almost one-third of the patients [7].

Here we present clinical and molecular characterization of a new case of de novo complex small supernumerary marker chromosome (sSMC) consisting of the entire short arm of chromosome 18p associated with a centromere of either chromosome 13 or 21, evidenced in a 5-year-old boy during work up for moderate intellectual disability and dysmorphisms.

To the best of our knowledge, the scientific literature reports about 35 cases of pure trisomy 18p, which may be originated by different chromosomal mechanisms: unbalanced translocations, duplications, deletion of 18p with an isochromosome 18p and supernumerary marker chromosomes, reviewed by Yu et al. [8].

To date, seven cases of isolated trisomy 18p due to a sSMC have been reported, three of which have been characterized by array CGH, in two of these the breakpoints and the size of the duplication are shown [9]. We reviewed similar cases reported in the DECIPHER database in order to identify patients carrying similar duplication [10]. We also considered smaller duplications within the region of interest, in order to evaluate the presence of critical regions implicated in the pathological phenotype.

Our case provides additional information about phenotypic effects of pure trisomy 18p, confirm chromosomal microarray analysis as goal standard to characterize complete sSMC, and supply additional elements for genetic counselling.

## Materials and methods

DNA was obtained from whole blood with MagCore Extractor System H16 using MagCore Genomic DNA Large Volume Whole blood kit (RBC Bioscience). Concentration and purity of DNA was verified with NanoPhotometer P-Class. Array CGH was performed using OGT (Oxford Gene Technologies) ISCA v2 4 × 180 K Microarray Kit according to the manufacturer’s instructions. Patient’s and the reference male DNA (Promega 147A) were labeled and hybridized using enzymatic labeling and hybridization protocols (OGT reagents). The image of the array was acquired using an Innoscan 710 Microarray scanner (Innopsis) and results were analyzed by Cytosure Interpret Software (v.4.8, Oxford Gene Technologies) using standard algorithms. FISH analysis was performed on lymphocyte metaphase spreads of the proband using specific chromosome 13/21 α-satellite D13Z1/D21Z1 centromeric probes (Kreatech) and whole chromosome painting probes specific for chromosome 18 (Kreatech) according to manufacturer’s recommendation.

## Case presentation

The patient was initially referred to a child neuropsychiatry unit at the age of 5 for mild global development delay, especially in the speech area. He is the youngest of three males of healthy non-consanguineous Caucasians parents, (the mother was 35 years old, the father 42 at conception). He was born at 39.2 weeks of spontaneous and unremarkable pregnancy, birth weight was 3410 gr (25–50th percentile), length 51 cm (50–75th percentile), HC 34 cm (25–50th percentile). At delivery monolateral club foot was evident and corrected with plaster casts worn for 3 months. At birth he was normal. During instrumental work-up brain MRI was performed and the presence of type 1 Arnold Chiari malformation without hydrocephalus, syringomyelia or fibrolipoma of the terminal filum was evidenced. Gastroenterological evaluation was also performed for frequent episodes of vomiting, concomitantly with upper respiratory tract infections. At X-ray analysis during transit neither gastroesophageal reflux nor vomiting episodes were reported.

At the last follow-up at the age of 7 physical examination indicated a height of 117,8 cm, weight of 25 kg and head circumference of 51 cm (25–50th percentile for all values) while neurological examination evidenced slight reduction in muscle tone in all districts and embarrassment of fine and global motor performance. Cognitive assessment performed by WISC IV scale indicated total IQ of 44 (VCI = 48, PRI = 67, WMI = 61, PSI = 56, GAI = 52, CPI = 48).

The boy has a regular feeding but he is still followed by clinicians of the gastroenterology unit, his sleep–wake rhythm is regular, has completed the second class of the primary school with personal support teacher, he willingly goes to school, relationship with other children and involvement in daily activities are good.

## Results

**Fig. 1:**
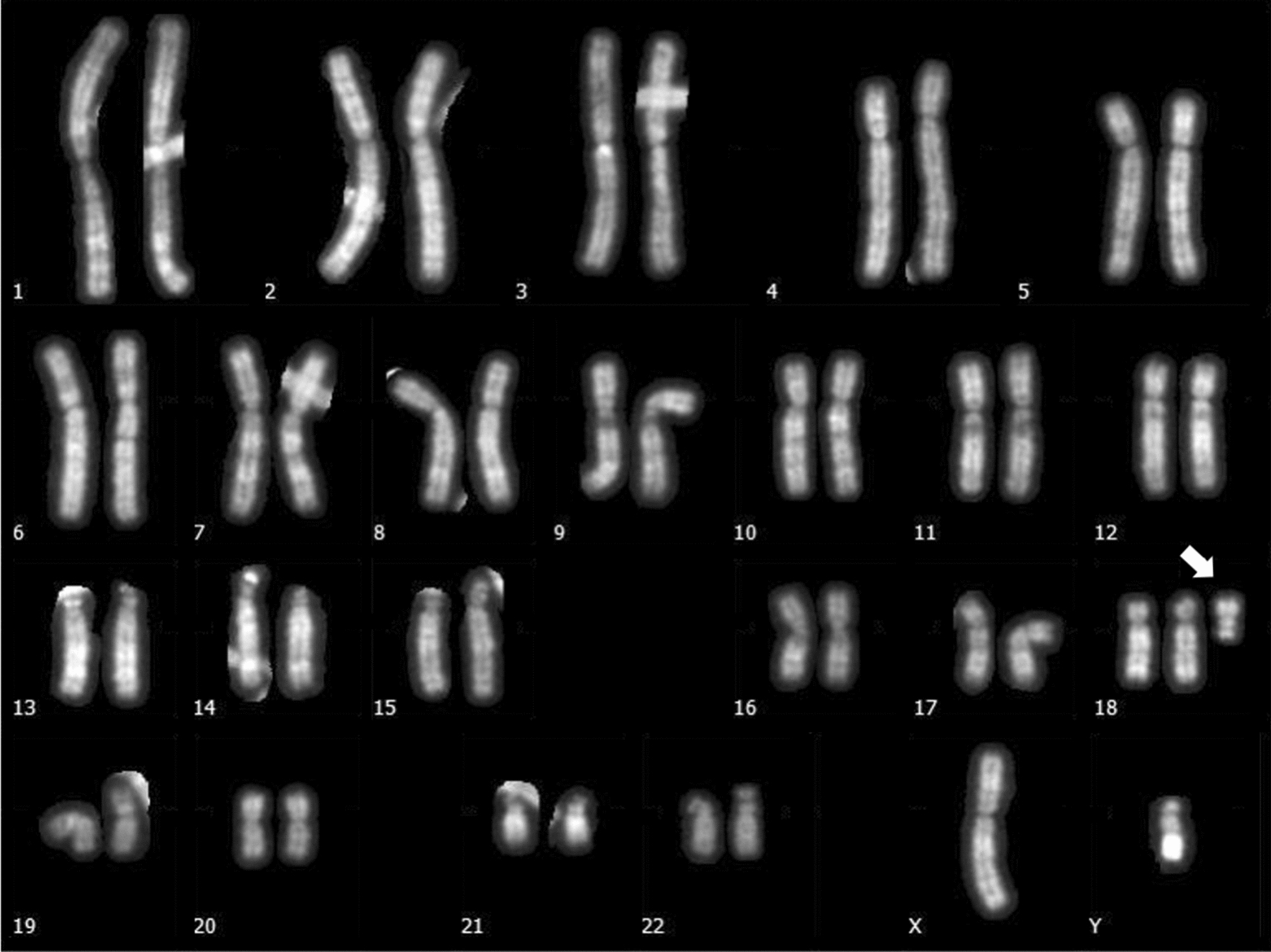
Metaphase of peripheral blood of the proband showing the presence of a supernumerary marker chromosome (white arrow). Cytogenetic analysis was performed using Q-banding at 550 bands resolution, in line with the International System for Human Cytogenetic Nomenclature (ISCN 2016)

Chromosome analysis was performed from cultured peripheral blood lymphocytes using high resolution QFQ banding (550 bands) [11]. The patient showed an abnormal male karyotype: 47,XY+mar, with a satellite sSMC in all the 50 metaphases analyzed (Fig. 1). Peripheral blood karyotype analysis performed on both propositus’ parents was 46,XX for the mother and 46,XY for the father, in all the 100 metaphases analyzed, indicating that sSMC occurred de novo.

**Fig. 2:**
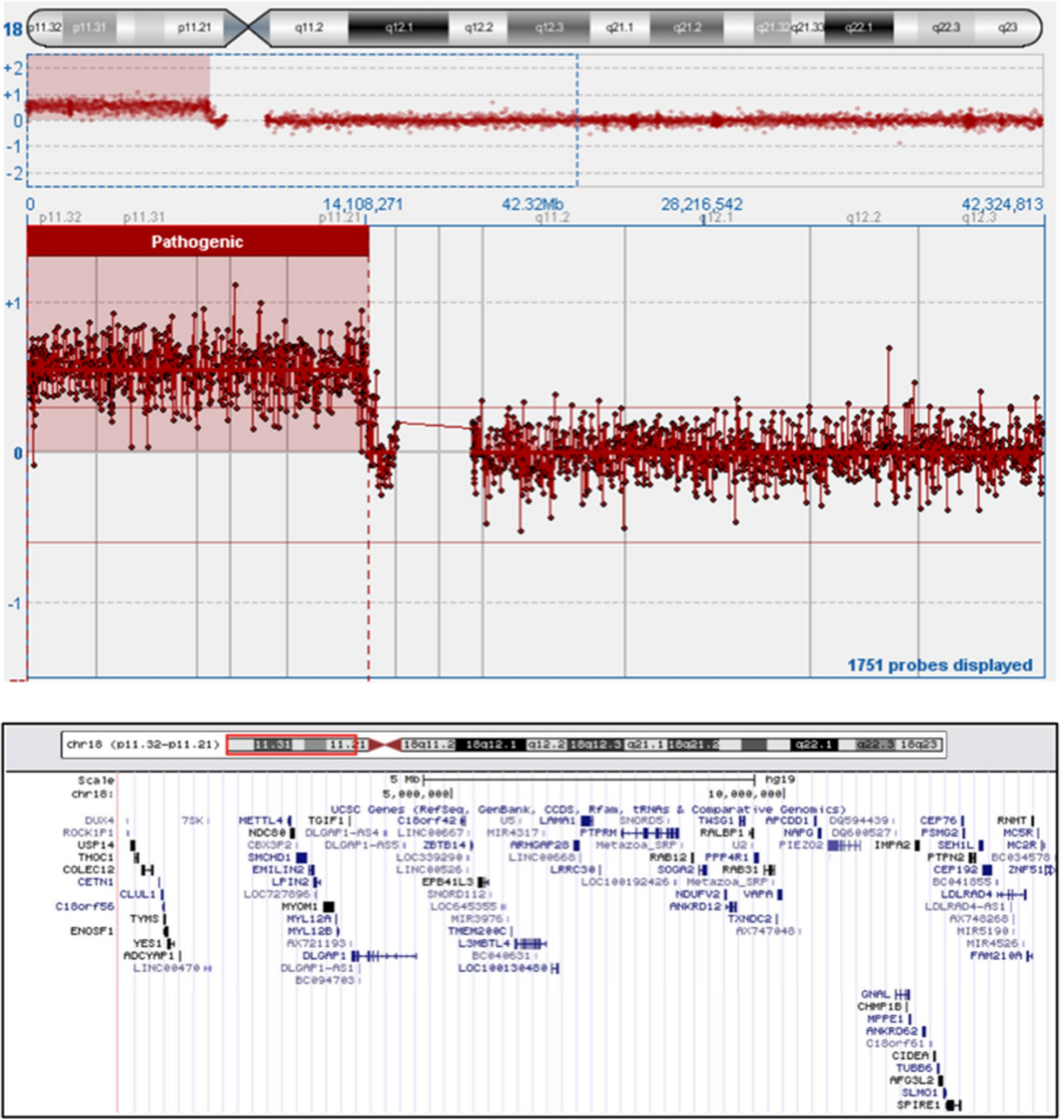
Array-CGH analysis: ideogram of chromosome 18 and dot plot of the duplication in 18p11.32p11.21, highlighted in the pink box (upper panel). Detail of the duplicated region spanning about 14 Mb as shown in the UCSC Genome Browser (lower panel). A number of piRNA genes present in the region has been omitted. The chr18:14,257-14,122,546 coordinates are relative to the GRCh37/hg19 assembly

**Fig. 3:**
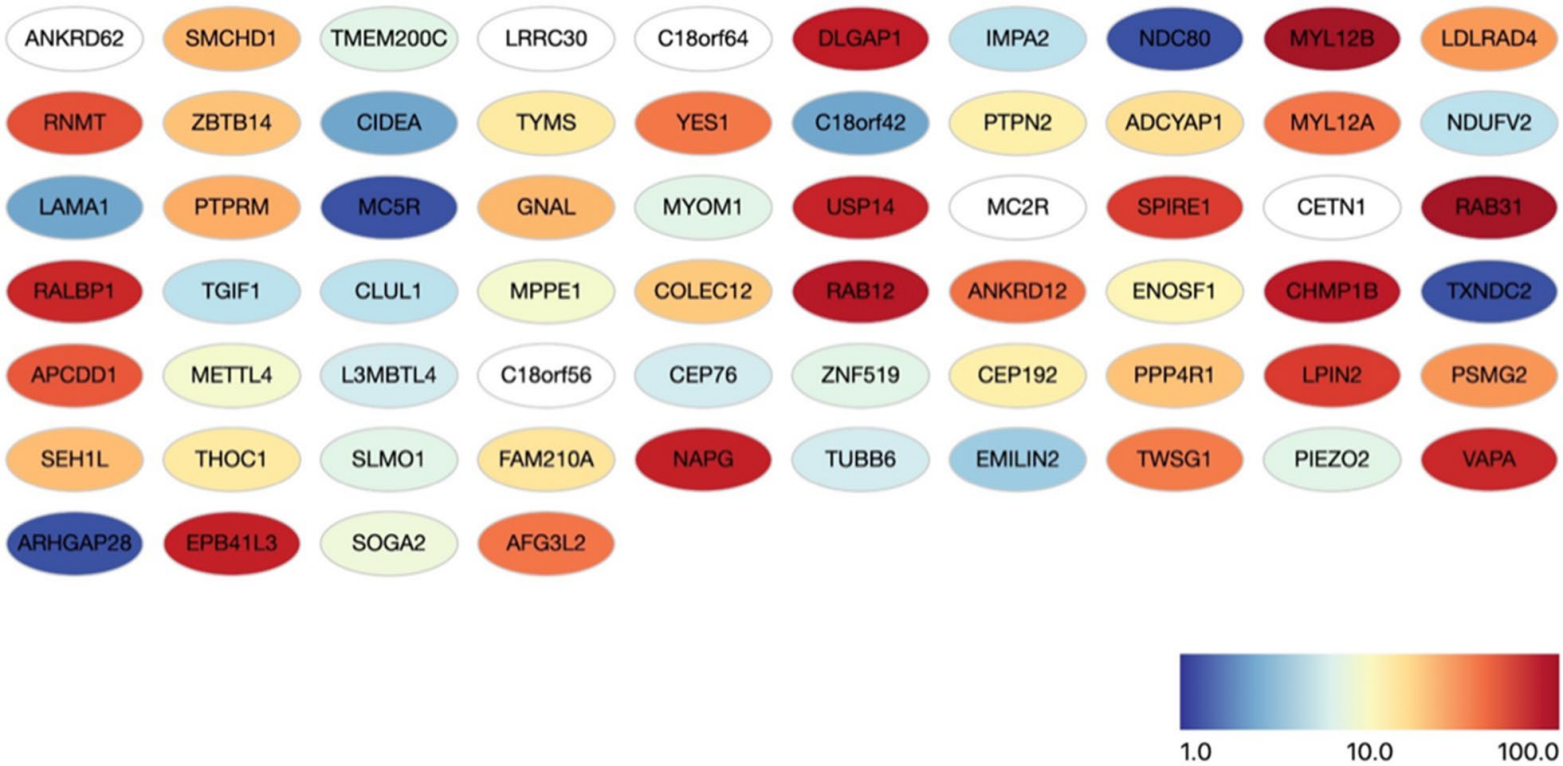
Expression levels in human cerebral cortex of the 64 protein coding genes present in the trisomic region. Genes are colored by expression (from blue, low expression to red, high expression). The analysis has been performed using Gonet (https://tools.dice-database.org/GOnet) [22], a bioinformatic tool that allows to retrieve data from the Human Protein Atlas db at https://www.proteinatlas.org

Array CGH was performed using the Oxford Gene Technology 4x180K platform revealed a duplication of the short arm of chromosome 18 spanning about 14,1 Mb, from 14257 bp (18p11.32) to 14122546 bp (18p11.21) (Fig. 2). It compasses 123 genes, including 64 protein coding genes. Interestingly a number of them is highly express in cerebral cortex (Fig. 3).

**Fig. 4:**
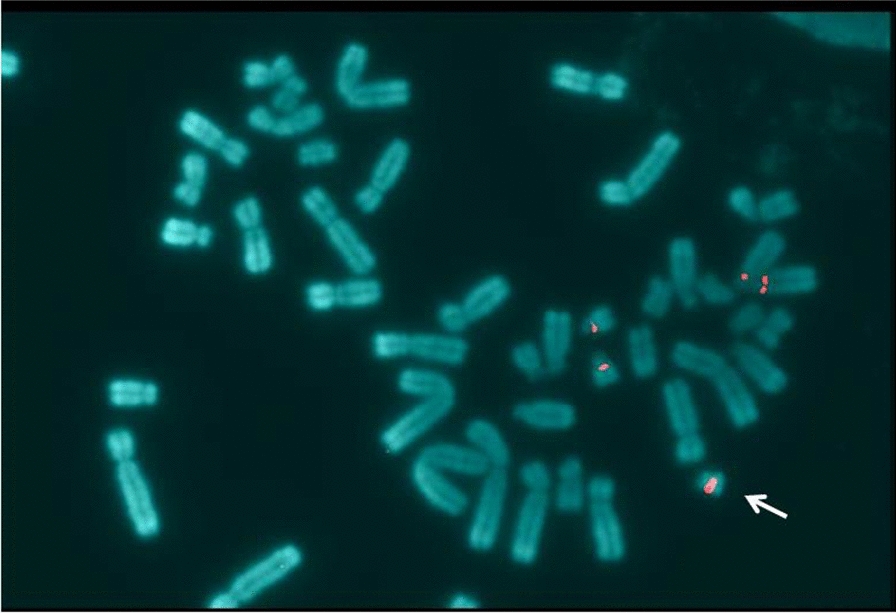
FISH analysis performed with D13Z1/D21Z1 centromeric probes (Kreatech™) which hibrydize both chromosomes 13 and chromosomes 21 centromeres. The image shows positive signals (labeled in red) on 13 and 21 omologues and an additional signal on sSMC (white arrow)

**Fig. 5:**
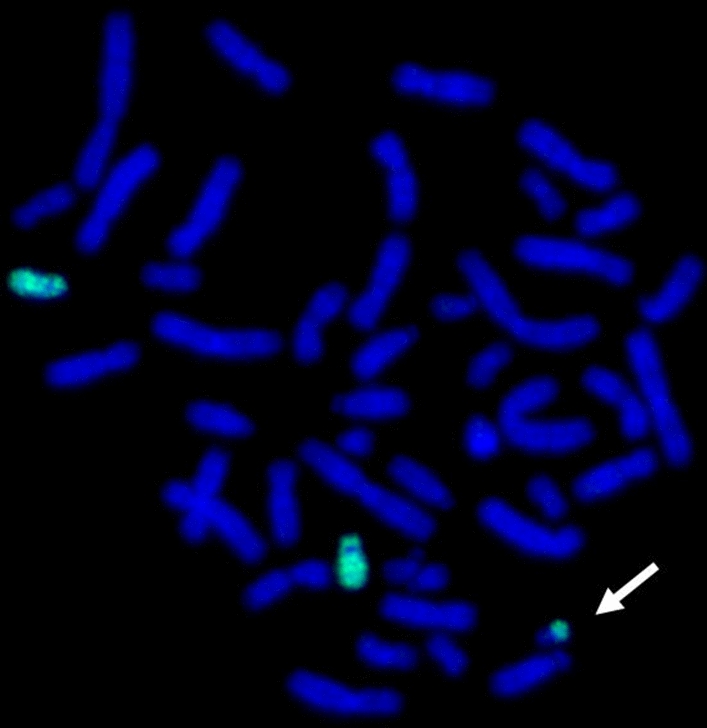
FISH analysis with whole chromosome 18 painting probes. The probes partially recognized the sSMC (white arrow) and confirmed the Array-CGH result showing that the duplicated 18p11.32p11.21 region is located in the sSMC

FISH analysis was performed on lymphocyte metaphase spreads of the propositus using specific chromosome 13/21 α-satellite D13Z1/D21Z1 centromeric probes. The analysis showed the presence of five hybridization signals corresponding to two chromosome 13 centromeres, two chromosome 21 centromeres and the centromere of sSMC (Fig. 4). In addition, the sSMC was partially hybridized by the whole chromosome 18 painting probes (Fig. 5). The final karyotype according to International System for Human Cytogenetic Nomenclature (ISCN 2016) is:

47,XY,+mar dn.ish der(13 or 21)t(13 or 21;18)(q11;p11.21)(D13Z1/D21Z1+,wcp18+).arr[GRCh37]18p11.32 p11.21(14257_14122546)x3

## Discussion and conclusion

Trisomy 18p is a rare chromosomal aberration usually not associated to a characteristic facial appearance with a cognitive spectrum ranging from normal intelligence to moderated intellectual disability. To the best of our knowledge there are six cases with pure 18 trisomy associated to sSMC. (Resumed in Table 1). Five of these consist of the entire short arm of chromosome 18 translocated to a chromosome 13/21 centromere [9, 12, 13]: three are de novo while the other two are familial. Sheth et al., [14] describe another sSMC derived from 18p but data on the origin of the centromere is missing. We describe a boy with a de novo trisomy of the entire short arm of chromosome 18 due to a sSMC originated from a complex rearrangement involving chromosome 18 and 13/21 centromeres. He shows moderate intellectual disability congruently with previously reported case with similar chromosome anomaly [9, 12, 13]. The trisomic region includes 64 protein coding genes and it is reasonable to hypothesize that the over-expression of some of them is responsible for the phenotypic abnormalities observed in the proband. Interestingly a number of them are highly expressed in cerebral cortex, indicating a significant biological role in CNS (Fig. 3). 11 of the genes present in triple copy are mutated in genetic disorders according to the OMIM database.

**Table 1:** Demographics data, phenotypes, molecular techniques used for characterization of sSMC: FISH (probes used indicated) and/or CGH or CGH array

	1. Mabboux et al. [12]	2. Rodriguez et al. [13]	3. Sheth et al. [14]	4. Plaja et al. [9] Patient 1	5. Plaja et al. [9] Patient 2	6. Present case
Final karyotype	47,XY,+mar.ish der(13/21)t(13/21;18)(q10;p11.21)	47,XY,+mar.ish der(13/21)t(13/21;18)	47,XX,+mar	47,XX,+der (13)t(13;18)(p11;q11.2)-13,+der(13;15)(p11;q11.1)	47,XY,+der(13)t(13;18)(p11;11.2)	47,XY,+mar.arr(18p11.32p11.21)(14257-14122546 bp) X3
Origin	De novo	Maternal (mother with mild mental retardation)	De novo	De novo	De novo	De novo
Sex	M	M	F	F	M	M
Age	13 years	2 months	13 years	17 years	V month of pregnancy	7 years
Phenotype	Mild mental retardation, mild face dysmorphic features, bilateral cryptorchidism	Club foot, defect in the atrial septum	Microcephaly, psychomotor delay, hypotonia followed by hypertonia, apnea, epilepsy hypertelorism, clinodactyly.	Non syndromic moderated intellectual disability	Single umbilical artery	Cognitive and speech delay
FISH performed and result ± of signal on SMC	WCP 13,18, 18p,21 13/21ɒsat 18 ɒ sat;BAC clones 18p;BAC clone13q; BAC clone 21q; PAC probe per NOR; subtel 18p Dj1174A5+,D13Z1/D21Z1+,D18Z1-,wcp18+,wcp18p+,D18S552+	Multicolour FISH 13/21ɒsat,18 ɒsat subcen13, subcen21 subbcen18 D13Z1+D21Z1+D18Z1-sbcen13- subcen21-subcen18+	Not performed	PW and Angelman subtel 15, centr 15,13/21 centr, 14/22 centr, centr 18, D13Z1/D21Z1+, D18Z1-, centr15-	PW e Angelman, 13/21 cent,18 centr. Spec. D13Z1/D21Z1+, D18Z1-	D13Z1/D21Z1+
Whole-genome array platform	CGH	no	Array CGH 4X 44 K	Array CGH 4X180K (SureprintG3 Human)	Array CGH 8X60K	Array CGH 4X180k
Size of duplication	Complete 18p trisomy	Complete 18p trisomy	15 Mb From 18pter to 18p11.21	13,98 Mb From 118760 bp (18p11.32) to 14102527 bp (18p11.21)	14,9 Mb From 14316 bp (18p11.32) to 14928854 bp (18p11.21)	14,11 Mb From 14257 bp (18p11.32 to 14122546 bp (18p11.21)
Mentioned genes involved in the trisomy	–	*MC2R, TYMS, LAMA1, YES1, NADH, NDUFV2, PTPN2, ERV1*	67 genes	44 genes, from *USP14* (158483 bp) to *MC2R* (13915535 bp)	44 genes, from *USP14* (158483 bp) to *MC2R* (13915535 bp)	123 HGNC genes. 64 are protein-coding genes

Size of the duplication and genes involved in this case and in the others cases of trisomy 18p due to sSMC reported in literature

Plaja et al. [9] have described two cases of sSMC derived from 18p with 13/21 centromeres: the first found in a 17 years old girl with non-syndromic intellectual disability and the second found during prenatal investigations in a 36 years old woman who underwent to amniocentesis for advanced maternal age. In the last case pregnancy was interrupted in consideration of fetal karyotype. Both patients show duplications of almost entire short arm of chromosome 18 spanning 13,98 Mb and 14,9 Mb respectively with breakpoints located within the 2 Mb pericentromeric region of 18p. In a previous study reporting the same anomaly only FISH and/or CGH analysis were performed [12, 13] and the size of duplication is missing. Our case shows a duplication of almost entire short arm of chromosome 18 spanning 14,1 Mb with a 1,3 Mb pericentromeric region showing a normal diploid state. On the basis of previously mentioned studies on similar cases we also performed 13/21 α-satellite FISH analysis that showed the presence of positive signal on the centromere of sSMC. FISH assay does not allow to distinguish between centromeres of chromosome 13/21 because the α-satellite subfamilies from chromosomes 13 and 21 are almost identical in sequence [15]. The duplicated region present in the sSMC of our proband encompasses 123 genes, 64 of which are protein coding.

Analyzing the DECIPHER database (http://decipher.sanger.ac.uk) [10] we found 67 patients with a duplication in 18p as single variant: in these patients, whose age is not reported, 5 duplications are de novo, 22 are inherited and in the remaining 40 cases the inheritance is unknown. The size range goes from 60,35 Kb to 15,29 Mb, most duplications occur either in 18p11.32 or in p11.31 bands.

6 patients carry duplications of unknown origin slightly larger than ours ranging from 14,59 to 15,11 Mb (vs 14,11 Mb) showing in addition to intellectual disability hypotonia and/or ataxia. These duplications encompass from a minimum of 14 to maximum of 20 of additional genes located on the centromeric side of the *ZNF519* gene (see Fig. 2) however, among these genes only 2, *ANKDR30B* and *POTEC*, are coding protein involved in breast and ovarian cancer respectively [16, 17]. Further studies will be needed to understand if and why these genes are involved in the more severe phenotype of the patients.

DECIPHER data seem to provide evidence about the role of small 18p duplications in worse clinical effects than the whole duplication. Beside intellectual disability, patients show other severe clinical features such as autism, abnormality of cardiovascular system morphology.

Deletions usually have serious effects while the interpretation of duplications is often a challenge and their role on the phenotype remain largely unresolved. If a gene at the ends of the duplication is broken, the phenotype of the subject could be comparable to haploinsufficiency. Moreover, a duplicated region could be inserted into another gene, but this does not happen in our subject.

In the literature there are some reports of interstitial microduplication that partially overlap each other with some genes in common. Giordano et al. [18] describe a microduplication of 320–431 Kb at 18p11.31-p11.23 identified through array CGH encompassing three genes (*ARHGAP28*, *LINC00668* and *LAMA1)* in 10 years old boy with moderate psychomotor delay and other physical anomalies: cerebellar vermis hypoplasia, coloboma, deafness and GH deficiency.

DECIPHER patient ID 25404 carry a duplication of 1,11 Mb at 18p11.31-p11.23 showing clinical features similar to those described by Giordano et al. [18]: aplasia/hypoplasia of cerebellar vermis, intellectual disability moderate sensorineural hearing impairment. The duplicated region compasses only five genes including *LAMA1*, a gene mutated in Poretti-Boltshauser syndrome, a neurodevelopmental disorder presenting congenital cerebellar anomalies and characterized by delayed motor development with cognitive function ranging from normal to intellectually disabled [19]. It is tempting to speculate that *LAMA1* may be a dosage-sensitive gene, considering that both loss of function mutations and duplications result in cerebellar alterations.

Balasubramanian et al. [20] report a family where father and two daughters of 6 and 5 years carry the same duplication in 18p11.32-p11.31 characterized by array CGH. This duplication is 2,6 Mb in size and include 9 to 13 HGNC genes. All patients show variable levels of intellectual disability, development delay and behavior difficulties without any physical anomalies variation except for microcephaly found in both siblings.

Kashevarova et al. [21] report on 8 years old boy with motor stereotypy, dysarthria, ADHD, autism and dysmorphic traits that carry a duplication of 350 Kb in 18p11.32 region inherited from his apparently healthy father.

Why some subjects with specific genomic imbalance present intellectual disabilities and other abnormalities while in others the same imbalance is without clinical effect is not clear. Further study will be needed to understand also the role of environmental factors on phenotype.

The case here presented together with those previously reported supports the mild phenotype effect associated to the duplication of entire 18p. Extensive follow-up is essential to provide adequate genetic counseling when the same chromosomal anomaly is found in prenatal diagnosis.
